# Development of an Innovative *Galleria mellonella* Model for Ricin Poisoning

**DOI:** 10.3390/toxins18060266

**Published:** 2026-06-12

**Authors:** Annabelle Garnier, Emilie Tessier, Arnaud Avril, Clémence Rougeaux

**Affiliations:** Unité Interaction Hôte-Pathogènes, Institut de Recherche Biomédicale des Armées, 91220 Brétigny sur Orge, France; annabelle.garnier@intradef.gouv.fr (A.G.); emilie.tessier@intradef.gouv.fr (E.T.); arnaud2.avril@intradef.gouv.fr (A.A.)

**Keywords:** ricin, *Galleria mellonella*, cultivars, poisoning model, therapeutic screening model

## Abstract

Ricin is considered a chemical and biological weapon. It is found in the seeds of a plant, *Ricinus communis*. There are different isoforms of ricin with different levels of toxicity, depending on the *R. communis* plant. There is no current prophylaxis and specific treatment for ricin poisoning is recent. Assessing potential countermeasures against this toxin still relies on testing on vertebrate animal models. *Galleria mellonella* larva, already used for the testing of bacteria, viruses, and fungi, represents an alternative model that is more ethical, inexpensive, and easier to use. In this study, we demonstrated for the first time the sensitivity of *G. mellonella* larvae to different cultivars of ricin. We observed mortality and a reduction in health index scoring over the days of testing. The health index scoring was based on the survival, the melanization, the mobility, and the capacity of larvae to produce a cocoon or not. Mortality was cultivar- and dose-dependent. Mortality of *G. mellonella* larvae was reduced when they were treated with a monoclonal antibody and concomitantly injected with ricin. Thus, *G. mellonella* represents a rapid and simple model of ricin poisoning, and, more particularly, a relevant model to test new therapeutics against ricin.

## 1. Introduction

Ricin, extracted from *Ricinus communis* (*R.c.*) castor beans, is one of the most potent poisons known (for a review, please refer to [1]). Due to its toxicity, ease of accessibility, readily available and inexpensive extraction from the castor bean plant, and the lack of specific medical countermeasures, ricin is classified as a List 1A compound in the Chemical Weapons Convention (CWC) and as a category B biological weapon by the Biological and Toxin Weapons Convention (BTWC). Thereby, the use of ricin as a biowarfare agent is ancient and recurrent [2,3,4,5,6,7,8,9]. In November 2025, three terrorists planning an attack with ricin were arrested in India [10]. Ricin also poses a public health problem with accidental poisonings and suicide attempts [1,11,12,13,14].

The toxin is a type 2 ribosome-inactivating protein (RIP), composed of an A-chain subunit (RTA) and a B-chain subunit (RTB), which are linked by a disulphide bond. RTB facilitates the binding and entry of ricin into the cytosol and its retrograde transport into cells [15,16,17]. RTA presents rRNA N-glycosylase activity that inactivates the 60S ribosomal subunit, preventing protein synthesis [18]. A single molecule of ricin is able to inactivate 1000 to 1500 ribosomes per minute.

Depending on the *R. communis* cultivar, there are different isoforms of ricin, in different proportions, with different levels of toxicity [19,20,21,22,23,24,25]—the two main ones being ricin D and ricin E [26]. Ricin D is present in all cultivars, while ricin E is not [25,27,28,29].

There are a few human cases out of several million that have occurred from plant poisoning, the majority of poisonings being accidental or associated with suicide [23]. Toxicity and symptoms depend on route of entry [16]. A fatal dose is estimated to range from 5 to 25 μg·kg^−1^ body weight for inhalation or injection exposure in human adults, and from 1 to 20 mg of ricin·kg^−1^ for oral toxicity. In mice, the lethal dose 50 (LD_50_) is 3 to 5 μg·kg^−1^ for inhalation exposure. For the parenteral route—which in history was used to assassinate a Bulgarian dissident [30]—the smallest LD_50_ is less than 1 μg·kg^−1^ in mice [16]. The LD_50_ after ingestion is evaluated to be 22–25 mg·kg^−1^. Clinical signs appear within a few hours, and death occurs within a few days. Very recently, an antidote for the treatment of acute intoxication with ricin, Ricimed, has been approved, requiring prompt administration [31]. The available symptomatic treatment only reduces the indirect effects of poisoning.

Assessing potential countermeasures against biological hazard agents relies on testing on animal models. To avoid the ethical, cost, and logistical constraints associated with the use of vertebrate animals, *Galleria mellonella* larva represents an alternative as it is already used for the testing of bacteria, fungi, and viruses [32,33,34]. This model has not yet been evaluated with toxins such as ricin, so we studied the sensitivity of *G. mellonella* larvae to ricin. As the amount and composition of ricin isoforms may vary, depending on the cultivar, we tested different cultivars of ricin injected into the haemocoel of larvae. A previously proposed health index scoring system was used to determine the health status of the *G. mellonella* [35,36]. It is based on monitoring four major criteria: the production of cocoon, larval melanization, mobility, and mortality. Melanization is a part of the innate and humoral defense system of *Galleria mellonella* [35,37] in response to microbial invasion. The melanin produced is deposited on the pathogen and sequesters it. Thus, we used this system to determine the effects of ricin. In this study, for the first time, we demonstrated a time-dependent sensitivity of *G. mellonella* to ricin, with differences between the cultivars tested. As determined for different pathogens, injection of ricin induced melanization in larvae, independently of the time, the cultivar, and the dose injected. We also observed a dose-dependent action. Finally, *G. mellonella* larvae were protected from ricin poisoning when they were treated with a ricin antibody. Based on these outstanding results, the *G. mellonella* model could be used instead of mice for the development of new therapies for ricin poisoning.

## 2. Results

### 2.1. G. mellonella Is Sensitive to Different Cultivars of Ricin

**Figure 1:**
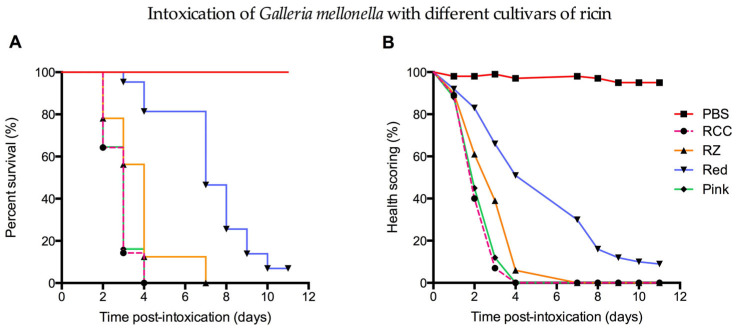
Percent survival (**A**) and health scoring (**B**) of *G. mellonella* injected with different cultivars of ricin. Larvae of *G. mellonella* were injected with 5 μg of ricin in 10 μL of PBS, as described in Section 4. We tested 4 cultivars: *R.c. Communis*—RCC; *R.c. Carmencita* Red—Red; *R.c. Carmencita* Pink—Pink; *R.c. Zanzibarensis*—RZ. Larvae injected with 10 μL of PBS were used as the negative control. Survival monitored over 11 days is expressed as percent survival and presented as Kaplan–Meier curves (**A**). The production of melanization, cocoon formation, and mobility were monitored over 11 days. Health index scoring was established with all the monitoring results and expressed as a percentage of health scoring (**B**). A total of 10 to 12 worms were assigned to each condition. Each experiment was repeated at least three independent times.

To characterize *G. mellonella* as an intoxication model for ricin, we tested different cultivars of ricin. We injected 5 μg of the different cultivars. PBS was used as a negative control. Larvae were monitored over 11 days for mortality, mobility, cocoon formation, and melanization (Figure 1).

**Figure 2:**
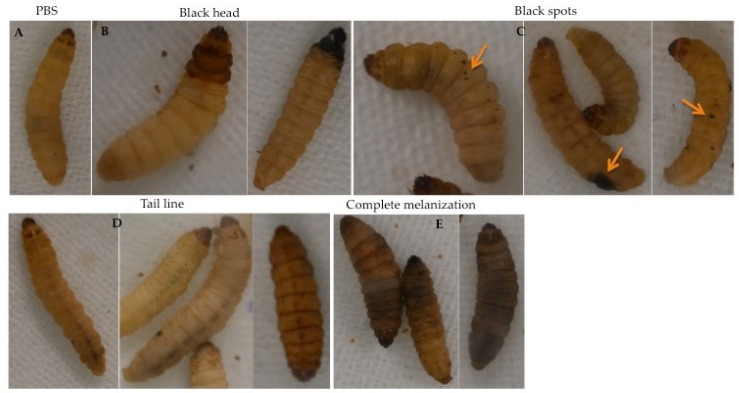
Intoxication of *Galleria mellonella* induces different types of melanization. Larvae were injected with PBS or 5 µg of different cultivars of ricin (10 µL in PBS): RCC, Pink, Red, and RZ. Larvae were monitored over 11 days. We observed creamy larvae (**A**) for the control PBS, and different types of melanization under intoxication: black head (**B**), small or big black spots ((**C**), the orange arrows highlitght different black spots), tail line (**D**), or complete melanization (**E**).

During the monitoring, PBS did not induce any mortality (Figure 1A), did not affect mobility, and the larvae remained cream-colored (Figure 2A).

Two days after injection, we observed the first deaths with *R.c.* Communis (RCC), *R.c.* Zanzibarensis (RZ), and *R.c.* Carmencita Pink (Pink). Mortality was observed three days after injection with *R.c.* Carmencita Red (Red) (Figure 1A). RCC and Pink were the most lethal, with 100% of mortality in 4 days and a median survival of 3 days (Table 1). Red was the less-lethal cultivar, with a median survival of 7 days and a survival proportion of 7%. Survival curves were significantly different (Log-rank (Mantel–Cox) test, *p* < 0.0001).

In addition to mortality, we monitored melanization. Intoxicated larvae displayed different types of melanization: black head of larvae (Figure 2B); black spots of varying sizes on larvae (Figure 2C); presence of a tail line (Figure 2D); or complete melanization (Figure 2E). The type of melanization was independent of the cultivar injected, independent of time, and started to be observed from 1 day post-injection for the four cultivars. For some larvae, the type of melanization evolved. A larva with a tail line could present later with complete melanization. This evolution was also independent of the cultivar injected. Larvae displaying melanization died, but we also observed creamy larvae that also died.

**Figure 3:**
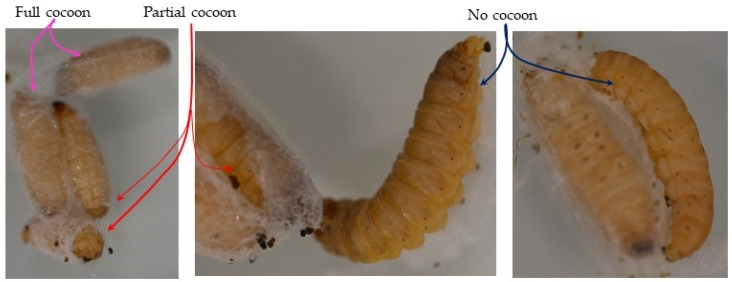
Intoxication of *G. mellonella* affects cocoon formation. Larvae were injected with PBS or 5 µg of different cultivars of ricin (10 µL in PBS): RCC, Pink, Red, and RZ. Larvae were monitored over 11 days. We observed full cocoons, partial cocoons, or no cocoons over the time of poisoning.

Intoxication also affected the formation of cocoons across the four cultivars (Supplementary Table S1). We observed partial cocoons and an increasing number of larvae without cocoons over the time of poisoning (Figure 3). Cocoon formation was slightly reduced for the PBS condition (79% of cocoon formation was the smallest value obtained, 9 days after challenge).

We established the evolution of the health index scoring for each cultivar with the results of melanization, cocoon formation, mobility, and mortality (Table 2, Figure 2B). None of the monitored criteria predominated over the others. As observed for the percent survival, the health index scoring of RCC and Pink rapidly diminished between day 0 and day 4 after injection. The health index scoring of RZ was broadly comparable, with a slight time shift. The health index scoring of Red declined more slowly between days 1 and 11 (Figure 2B).

### 2.2. Ricin Presents a Dose Effect

In an attempt to study a dose effect of ricin, larvae were injected with different doses of RCC and Pink: 3.75 μg, 2.5 μg, 1.25 μg, and 0.5 μg. We chose the two most toxic cultivars. We used the same batch of cultivar to avoid variations associated with the production of the toxin. Larvae were monitored over 11 days.

**Figure 4:**
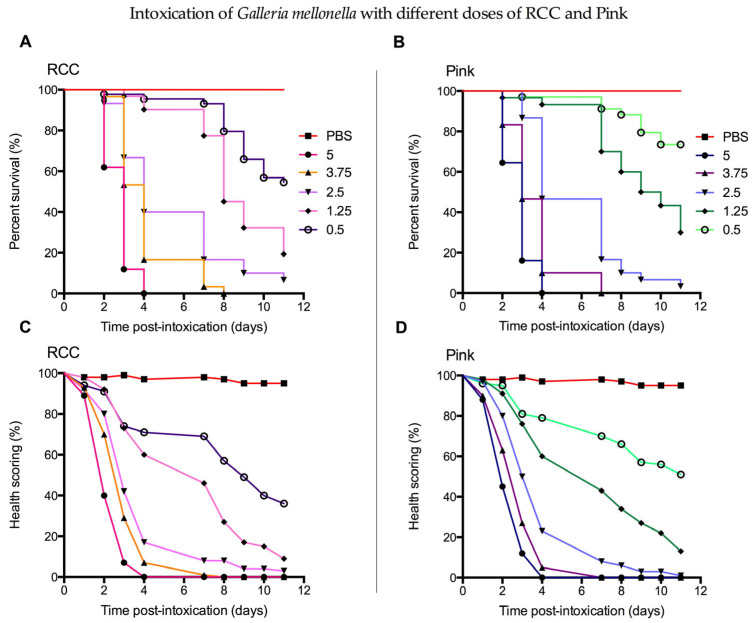
Percent survival (**A**,**B**) and health scoring (**C**,**D**) of *G. mellonella* injected with different doses of RCC (**A**,**C**) and Pink (**B**,**D**). Larvae of *G. mellonella* were injected with different doses of RCC and Pink in 10 μL of PBS, as described in Section 4. Survival was monitored over 11 days and expressed as percent survival. The production of melanization, cocoon formation, and mobility were monitored over 11 days. Health index scoring was established with all the monitoring results and expressed as a percentage of health scoring. A total of 10 to 12 worms were assigned to each condition. Each experiment was repeated at least three independent times.

For the two cultivars, there was a dose effect (Figure 4A,B). Survival was still 0% with 3.75 μg of RCC (Figure 4A). There was a weak survival rate of 6.7% at 2.5 μg that slightly increased to 19% with 1.25 μg of RCC. The percent survival was 55% after injection of 0.5 μg of RCC. The median survival increased to 4 days with 3.75 μg, and doubled to 8 days with 1.25 μg (Table 3).

Pink remained very effective at the highest doses tested, with a percent survival of 0% at 3.75 μg and only 3.3% with 2.5 μg (Figure 4B). The percent survival suddenly increased to 30% with 1.25 μg and 74% with 0.5 μg. The median survival was little changed, with 3 days for 5 μg and 3.75 μg and 4 days for 2.5 μg, while it was more elongated with 9.5 days at 1.25 μg (Table 3).

For RCC and Pink, the survival curves presented significant differences between them (Log-rank Mantel–Cox test, *p* < 0.0001). In our conditions and with these batches of toxins, LD_50_ was estimated at 0.56 μg for RCC (3.7 mg·kg^−1^) and 0.81 μg for Pink (5.4 mg·kg^−1^).

The health index scoring was calculated for each dose tested (Figure 4C,D). The health index scoring for RCC and Pink at 5, 3.75, and 2.5 μg displayed a similar profile, with a rapid decrease in their value. For both cultivars, values were inferior to 10% at 7 days after the challenge with 2.5 μg. At a dose of 1.25 μg, for RCC and Pink, the decrease was slower but the health index scoring was inferior to 20% over 11 days. Injection of 0.5 μg induced a slow decrease in the health index scoring, but it was more toxic for RCC, with a value of 36% over 11 days, while the value was 51% for Pink over the same time.

Moreover, the different doses of toxins did not affect the different patterns of melanization observed with 5 μg, and the formation of cocoons remained very reduced following the poisoning at different doses (Tables S2 and S3).

### 2.3. G. mellonella Is an Efficient Model to Test Treatment Against Ricin

To test the capacity of *G. mellonella* to be protected against ricin, we used a monoclonal antibody (mAb), 43RCA-G1. This antibody recognizes the two main isoforms, D and E [38,39].

**Figure 5:**
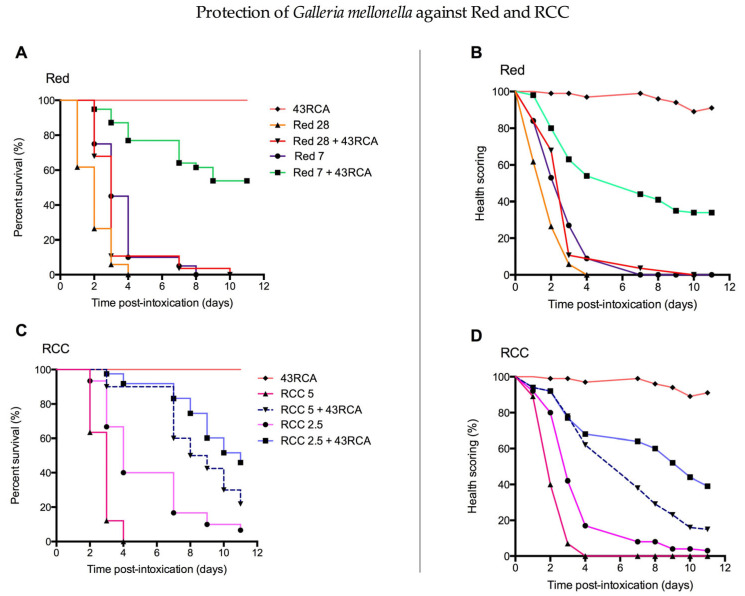
Percent survival (**A**,**C**) and health scoring (**B**,**D**) of *G. mellonella* injected with 43RCA-G1 antibody, Red (**A**,**B**), and RCC (**C**,**D**). Larvae of *G. mellonella* were concomitantly injected with 1 μg of 43RCA-G1 and different doses of *R.c.* carmencita Red or *R.c.* Communis, as described in Section 4. Injection of ricin cultivar alone was used as a control of poisoning. An amount of 1 μg of 43RCA-G1 was injected alone to test toxicity of the mAb. Survival was monitored over 11 days and expressed as percent survival. The production of melanization, cocoon formation, and larval mobility were monitored over 11 days. Health index scoring was established with all the monitoring results and expressed as a percentage of health scoring. A total of 10 to 12 worms were assigned to each condition. Each experiment was repeated three independent times.

Absence of toxicity was verified by the injection of 1 μg 43RCA-G1 alone (Figure 5A,C). The administration of the antibody did not induce mortality, and cocoon production was only slightly reduced in the later days.

To determine the protective effect of 43RCA-1, 1 μg of mAb was first injected, and 7 μg of Red was injected afterward into the larvae. Compared to the larvae poisoned with 7 μg of Red, we observed an important protection with the mAb, with a significant difference between the survival curves (Log-rank Mantel–Cox test, *p* < 0.0001). The percent survival increased to 54% (Figure 5A, Table 4), the health index scoring improved to a value of 34% (Figure 5B), and the formation of cocoons remained very reduced (Table S4). We tested the protection for a higher dose of Red, 28 μg. The injection of 28 μg of Red alone induced total mortality in 4 days (the health index scoring was 0%). The protection was drastically reduced, with a median survival of 3 days versus 2 days for larvae inoculated with 28 μg, and a percent survival of 2% at 9 days after challenge, before total mortality 10 days after intoxication (Figure 5A, Table 4).

We tested the protective effect of 43RCA-1 with another cultivar, RCC (Figure 5C,D). The protection was less important when 5 μg of RCC was injected: the median survival was prolonged to 8.5 days, but percent survival was 22% (Table 4), and the health index scoring remained low, with a value of 15% at 11 days after injection (Figure 5D). The protective effect was more pronounced for a dose of 2.5 μg of RCC. Median survival and survival proportion were increased to 11 days and 46%, respectively, compared to 4 days and 6.7% for larvae poisoned with 2.5 μg of RCC (Table 4). The health index scoring was slightly increased to 39% at 11 days after challenge (Figure 5D).

For both cultivars, the formation of cocoons remained very reduced, even with the antibody (Table S4).

## 3. Discussion

For the first time, we demonstrate the susceptibility of *G. mellonella* to ricin, a toxin of biological and chemical threat. The ease of use of this model allowed us to show that toxicity is time-dependent and varies according to the cultivar tested, as previously observed in vitro. In our study, the cultivar Red is the least efficient, with the highest median survival (7 days) despite a low survival proportion (7%) and a low health index scoring (5%). The cultivar Red contains isoforms D and E, as does Pink [25,29], which is as toxic as RCC, which also contains isoforms D and E, inducing total mortality in 4 days. Both cultivars induced a drastic and rapid reduction in mobility, cocoon formation, and induction of melanization. The cultivar RZ, which contains only the isoform D [28], presents a nearly similar level of toxicity. The difference in toxicity could be due to the important heterogeneity of ricin [23]: (i) there are several *R. communis* seed varieties; (ii) isoform composition and proportion differ among cultivars; (iii) isoforms present different levels of glycosylation that could affect their toxicity.

The presence of ricin D and E in Pink is not necessarily responsible for the high toxicity of this cultivar, as Red also displays these two isoforms. If ricin E seems less toxic than ricin D in the different cell lines tested, the combination of both did not induce greater toxicity [20,39,40]. The preparation can also contain different proteins of a given isoform, with different activities. Thus, an analysis revealed that *R.c.* Carmencita, Impala, Sanguineus, and Gibsonii cultivars have a similar ricin glycosylated protein content, different from that of the *R.c.* Zanzibarensis cultivar [25]. Another analysis highlighted that ricin D from the *R.c*. Sanguineus cultivar presents different isoforms, one of which is more toxic than the others, with a higher phospholipase activity [41]. In the studies of Sehgal, three isoforms of D were isolated from *R.c.* Communis seeds, an Indian variety: ricin I, II, and III. Ricin III is the most toxic, in vivo and in vitro [24,42]. This isoform is more rapid in generating reactive oxygen species (ROS) and oxidative stress, which is probably due to its high level of glycosylation [24]—the glycosylation affecting transport of RTA out of the endoplasmic reticulum [43]. In our study, the level of glycosylation of proteins contained in the different cultivars tested could explain the variable toxicity.

The purification step can influence the variability in toxicity. The impact on the toxicity of the purification protocol used to extract ricin was addressed in the review of Worbs et al. [23]. Obtaining pure ricin may lead to bias, as observed by Schieltz et al. [21]. In their study, they compared the N-glycosidase activity of 18 cultivars of *R. communis* using mass spectrometry; these cultivars did not have significantly different activity, but they displayed a higher specific activity than that of the ricin standard. An explanation is that purification of the ricin standard could have degraded its activity. We determined a lethal dose 50 (LD_50_) for two cultivars in this study, but we have to keep in mind that these values are valid only for the batches we used in our study. Therefore, results may vary depending on the batches produced and the conditions of preparation of the toxin. This is one limitation of the study. To be more rigorous, the tests could be realized with productions whose proportions of isoforms and glycosylation are characterized. Thus, it would be interesting to test ricin standard material to establish a reference LD_50_ [19,29]. Nevertheless, cases of ricin poisonings are often associated with preparations of highly variable quality.

The seeds of the *R. communis* plant contain an equivalent proportion of ricin proteins and another toxin, *R. communis* agglutinin (RCA) [21]. As ricin, RCA presents N-glycosidase activity, but its toxicity is lower than that of ricin by a factor of 50–2000 [44,45,46,47,48]. Thus, RCA, with a mass of 120 kDa, could be implicated in the toxicity of *G. mellonella*. The preparations of cultivars used in our study displayed a major band at 60–65 kDa under the non-denaturing condition (non-denaturing gel electrophoresis) and two major bands at ~35 kDa under denaturing conditions; therefore, the effects observed on the larvae are mainly due to ricin proteins.

In *G. mellonella*, we demonstrate that the toxicity also depends on the dose injected, with differences displayed between cultivars. RCC and Pink present a high toxicity up to a dose of 2.5 μg. Differences between the two cultivars appear at lower doses, with RCC being more effective than Pink. The dose-dependent toxicity has been previously demonstrated in different animal models of ricin intoxication [49,50,51,52].

Melanization is a well-established defense response in *G. mellonella* [35,37,53,54,55,56]. Herein, we observed that ricin intoxication can induce melanization, independent of the cultivar, the dose, and the time. Whether the melanin controlled the intoxication or not could be interesting to study.

*G. mellonella*, being an easy-to-use model and giving relatively rapid results, could be integrated into a combination of assays for the detection of active ricin in biological samples [44,57]. However, ricin levels found in fluids after poisoning are too low to be detected, and the sensitivity of *G. mellonella* is insufficient compared to immunodetection methods [3,58,59,60].

Finally, *G. mellonella* can represent an efficient model to test and screen anti-ricin drugs, as it has been used for antifungal and antibacterial drug evaluation [33,54,61]. Its use responds to the 3Rs principle (Replacement, Reduction, and Refinement) from Russell and Burch, which aims to minimize the employment of mice in research [62]. Until now, in vitro assays and vertebrate animals have been used to test anti-ricin drugs [39,58,63,64,65,66,67,68]. We demonstrate here that *G. mellonella* can be a powerful model for an intermediate screen of molecules, before the use of mice and non-human primates or macaques. Herein, we tested the humanized antibody 43CA-G1 that protects cell lines from poisoning with ricin and slightly improves mice survival, especially when injected concomitantly with ricin [39]. In our study, with this injection timing, we also observed the protection of larvae, providing a proof of concept. The level of protection depends on the cultivar of ricin and the dose of ricin. These parameters must be considered for future tests. It will also be interesting to test other treatment times to assess the level and duration of protection for the larvae. This will enable the investigation of whether delayed treatment administration decreases protection, as observed in mice with the antibody 43CA-G1 [39].

To conclude, our results offer promising perspectives for using *G. mellonella* as a model of ricin poisoning, particularly from the perspective of drug screening.

## 4. Materials and Methods

### 4.1. Cultivars of Ricin

The different cultivars of ricin were prepared and supplied by an external institute (DGA Maîtrise NRBC, Paris, France). The given quantities correspond to the quantity of ricin measured during preparation. The ricin was used in dedicated toxin facilities (BSL-2 level): *Ricinus communis* (RCC); *R.c.* Carmencita Red (Red); *R.c.* Carmencita Pink (Pink); *R.c.* Zanzibarensis (RZ).

RCC and RZ are commonly used in studies on ricin, while the other two are employed less frequently.

The cultivars RCC, Red, and Pink contain the isoform D and the isoform E. The cultivar RZ contains the isoform D.

### 4.2. Galleria mellonella Intoxication

*G. mellonella* larvae worms, weighing between 100 and 200 mg, were purchased from INRAE (Jouy-en-Josas, France). Ricin from the different cultivars was prepared to the desired concentration in PBS. A total of 10 to 12 worms were assigned to each concentration and kept in Petri dishes, without food. An amount of 10 μL of ricin was injected in the uppermost proleg, as previously described [69], using an infusion pump KDS 100Y (Delta Labo, Avignon, France), a 1 mL syringe (UGAP, Champs-sur-Marne, France), and a Venofix^®^ A 27 G set (UGAP, France). Larvae injected with 10 μL of phosphate-buffered saline (PBS) were used as a negative control. The tests were performed with larvae incubated at 37 °C in the dark. Surviving larvae at the end of monitoring were placed at −20 °C before their elimination. Each experiment was performed in triplicate.

### 4.3. Monitoring of Larvae

Wax worms were monitored over 11 days. To assess finer variations in larval health status, we examined in each larva 4 major criteria: melanization, cocoon formation, mobility, and survival (Table 2). Healthy larvae remained cream-colored and presented a full cocoon and movement without stimulation. Diseased larvae could present melanization. Diseased larvae could also present a partial cocoon or no cocoon, reduced mobility (no movement or movement on stimulation), or could be dead. A health index scoring was established from the results of observations of the 4 criteria for each larva; this was adapted and simplified from a previously established health index scoring [36]. The total score determined was expressed as a percentage. The value of 100% corresponds to healthy larvae at day 0, with cocoon formation, mobility, cream coloration, and an alive status (score of 8).

### 4.4. Protection of Larvae

The humanized 43RCA-G1 monoclonal antibody [38] was prepared at 0.1 mg.mL^−1^ in PBS. To assess its toxicity, 10 μL was injected in the last proleg, as described in Section 4.2 on *G. mellonella* intoxication. To assess its protective effect, 10 μL was injected in the last proleg, as described in Section 4.2 on *G. mellonella* intoxication, and 10 μL of Red and RCC was injected afterward, in the opposite proleg. Injected larvae were incubated at 37 °C, in the dark, and monitored over 11 days, as described in Section 4.3 on the monitoring of larvae. Each experiment was performed in triplicate.

### 4.5. Statistical Analysis

Each experiment was performed at least in triplicate. For each experiment, 10 to 12 larvae were assigned. For a given condition, data from each experiment were pooled for analysis and graphs. Analysis and graph generation were performed with GraphPad Prism software (version 4.0; GraphPad Software, San Diego, CA, USA). The survival of *G. mellonella* larvae was plotted as a survival curve using the Kaplan–Meier method. The analysis of survival curves with GraphPad Prism software provided the median survival and survival proportion. Statistical significance between survival curves was determined using the Log-rank (Mantel–Cox) test. The lethal dose 50 (LD_50_) values of *G*. *mellonella* larvae inoculated with different doses of RCC and Pink were determined using probit analysis with 95% confidence intervals (nonlinear regression analysis, GraphPad Prism software, version 4.0).

## Figures and Tables

**Table 1:** Median survival and survival proportion of *G. mellonella* injected with different cultivars of ricin.

Condition	PBS	RCC	RZ	Red	Pink
Median survival (days)	No mortality	3	4	7	3
Survival proportion (percentage)	100	0	0	7	0

Larvae of *G. mellonella* were injected with 5 μg of ricin in 10 μL of PBS, as described in Section 4. We tested 4 cultivars: RCC, Red, Pink, and RZ. Larvae injected with 10 μL of PBS were used as the negative control. Median survival and survival proportion were calculated with GraphPad Prism. A total of 10 to 12 worms were assigned to each condition. Each experiment was repeated at least three independent times.

**Table 2:** *G. mellonella* health index scoring system. Alive and healthy larvae present spontaneous mobility, full cocoon, and no melanization (score of 8).

Description	Score
No melanization	2
Melanization	0
Movement without stimulation	2
Movement upon stimulation	1
No movement	0
Full cocoon	2
Partial cocoon	1
No cocoon	0
Survival	2
Death	0

Larvae of *G. mellonella* were injected with ricin in 10 μL of PBS, as described in Section 4. Larvae injected with 10 μL of PBS alone were used as the negative control. The production of melanization, cocoon formation, mobility, and survival were monitored over 11 days. Healthy larvae were alive (score of 2), presented no melanization (score of 2), displayed mobility without stimulation (score of 2), and full cocoon formation (score of 2). Intoxicated larvae could be dead (score of 0), display movement upon stimulation (score of 1) or no movement (score of 0), display partial cocoon formation (score of 1) or no cocoon formation (score of 0), or display melanization (score of 0). Results allowed for the determination of a health index scoring.

**Table 3:** Median survival and survival proportion of *G. mellonella* injected with different doses of RCC and Pink.

	Dose (μg)	5	3.75	2.5	1.25	0.5
RCC	Median survival (days)	3	4	4	8	Undefined
	Survival proportion (percentage)	0	0	6.7	19	55
Pink	Median survival (days)	3	3	4	9.5	Undefined
	Survival proportion (percentage)	0	0	3.3	30	74

Larvae of *G. mellonella* were injected with different doses of RCC and Pink in 10 μL of PBS, as described in Section 4. Median survival and survival proportion were calculated with GraphPad Prism. A total of 10 to 12 worms were assigned to each condition. Each experiment was repeated at least three independent times.

**Table 4:** Median survival and survival proportion of *G. mellonella* treated with 43RCA-G1 mAb and injected with RCC and Red.

**Condition**	**43RCA-G1**	**Red** **28 μg**	**43RCA-G1 + Red 28 μg**	**Red** **7 μg**	**43RCA-G1 + Red 7 μg**
Median survival (days)	Undefined	2	3	3	Undefined
Survival proportion (percentage)	100	0	0	0	54
**Condition**	**43RCA-G1**	**RCC** **5 μg**	**43RCA-G1 + RCC 5 μg**	**RCC** **2.5 μg**	**43RCA-G1 + RCC 2.5 μg**
Median survival (days)	Undefined	3	8.5	4	11
Survival proportion (percentage)	100	0	22	6.7	46

Larvae of *G. mellonella* were injected with 10 μL of the humanized 43RCA-G1 monoclonal antibody at 0.1 mg.mL^−1^ in PBS. An amount of 10 μL of Red or RCC was injected afterward, as described in Section 4. Median survival and survival proportion were calculated with GraphPad Prism. A total of 10 to 12 worms were assigned to each condition. Each experiment was repeated three independent times.

## Data Availability

Data is contained within the article and Supplementary Material.

## Supplementary Materials

The following supporting information can be downloaded at: https://www.mdpi.com/article/10.3390/toxins18060266/s1, Table S1: Health index scoring for cocoon formation, obtained for *G. mellonella* intoxicated with different cultivars of ricin; Table S2: Health index scoring for cocoon formation, obtained for *G. mellonella* intoxicated with different doses of RCC; Table S3: Health index scoring for cocoon formation, obtained for *G. mellonella* intoxicated with different doses of Pink; Table S4: Health index scoring for cocoon formation, obtained for *G. mellonella* intoxicated with Red and RCC and protected with 43RCA-G1.

## Abbreviations

The following abbreviations are used in this manuscript:

RCC	*Ricinus communis* Communis
R.c.	*Ricinus communis*
RZ	*Ricinus communis* Zanzibarensis
Red	*Ricinus communis* Carmencita Red
Pink	*Ricinus communis* Carmentita Pink
RCA	*R. communis* agglutinin
RIP	ribosome-inactivating protein
RTA	A-chain subunit
RTB	B-chain subunit
LD_50_	lethal dose 50
