# Supplementary material for: Development of an Innovative Galleria mellonella Model for Ricin Poisoning
Source: Toxins (Basel). 2026 Jun 12;18(6):266. doi: 10.3390/toxins18060266 (PMC13308285; doi:10.3390/toxins18060266)
Supplement: Supplementary file 1 [file toxins-18-00266-s001.zip › toxins-4343620-supplementary.pdf]

Supplementary Materials: Development of an Innovative *Galleria mellonella* Model for Ricin Poisoning

Annabelle Garnier, Emilie Tessier, Arnaud Avril and Clémence Rougeaux *

This supplementary material contains 4 tables.

Table S1. Health index scoring for cocoon formation, obtained for *G. mellonella* intoxicated with different cultivars of ricin.

	**Day post intoxication**	**D0**	**D1**	**D2**	**D3**	**D4**	**D7**	**D8**	**D9**	**D10**	**D11**
**Cocoon formation (%)**	**PBS**	100	98	98	99	97	98	97	95	95	95
	**RCC**	100	85	35	5	0					
	**RZ**	100	75	42	14	0					
	**Red**	100	91	84	34	14	3	2	2	2	2
	**Pink**	100	73	33	8	0					

Table S2. Health index scoring for cocoon formation, obtained for *G. mellonella* intoxicated with different doses of RCC.

**RCC**	**Day post intoxication**	**D0**	**D1**	**D2**	**D3**	**D4**	**D7**	**D8**	**D9**	**D10**	**D11**
**Cocoon formation (%)**	**5 µg**	100	85	35	5	0					
	**3.75 µg**	100	83	60	12	0					
	**2.5 µg**	100	78	82	20	3	0				
	**1.25 µg**	100	93	23	3	0	0	0	0	0	3
	**0.5 µg**	100	86	77	26	7	11	5	3	6	5

Table S3. Health index scoring for cocoon formation, obtained for *G. mellonella* intoxicated with different doses of Pink.

**Pink**	**Day post intoxication**	**D0**	**D1**	**D2**	**D3**	**D4**	**D7**	**D8**	**D9**	**D10**	**D11**
**Cocoon formation (%)**	**5 µg**	100	73	33	8	0					
	**3.75 µg**	100	73	53	17	0					
	**2.5 µg**	100	87	72	13	2	0				
	**1.25 µg**	100	90	77	43	3	3	0	0	0	0
	**0.5 µg**	100	83	88	35	27	15	11	3	15	3

Table S4. Health index scoring for cocoon formation, obtained for *G. mellonella* intoxicated with Red and RCC and protected with 43RCA-G1.

**Cocoon formation (%)**	**Day post intoxication**	**D0**	**D1**	**D2**	**D3**	**D4**	**D7**	**D8**	**D9**	**D10**	**D11**
	43RCA-G1	100	100	100	100	97	100	95	85	87	92
**Red**	43RCA-G1 + Red 28 µg	100	78	14	0						
	43RCA-G1 + Red 7 µg	100	93	52	20	8	10	10	5	7	7
**RCC**	43RCA-G1 + RCC 5 µg	100	83	89	58	31	20	5	8	3	3
	43RCA-G1 + RCC 2.5 µg	100	85	80	30	23	26	23	23	23	16
